# Establishment and function of chromatin modification at enhancers

**DOI:** 10.1098/rsob.200255

**Published:** 2020-10-14

**Authors:** Amanuel Tafessu, Laura A. Banaszynski

**Affiliations:** UT Southwestern Medical Center, Cecil H. and Ida Green Center for Reproductive Biology Sciences, Department of Obstetrics and Gynecology, Children's Research Institute, Hamon Center for Regenerative Science and Medicine, Dallas, TX 75390-8511, USA

**Keywords:** enhancer, chromatin, post-translational modification, histone variants, phase separation, transcription

## Abstract

How a single genome can give rise to distinct cell types remains a fundamental question in biology. Mammals are able to specify and maintain hundreds of cell fates by selectively activating unique subsets of their genome. This is achieved, in part, by enhancers—genetic elements that can increase transcription of both nearby and distal genes. Enhancers can be identified by their unique chromatin signature, including transcription factor binding and the enrichment of specific histone post-translational modifications, histone variants, and chromatin-associated cofactors. How each of these chromatin features contributes to enhancer function remains an area of intense study. In this review, we provide an overview of enhancer-associated chromatin states, and the proteins and enzymes involved in their establishment. We discuss recent insights into the effects of the enhancer chromatin state on ongoing transcription versus their role in the establishment of new transcription programmes, such as those that occur developmentally. Finally, we highlight the role of enhancer chromatin in new conceptual advances in gene regulation such as condensate formation.

## 1. Introduction

Enhancers are short (typically 100 bp to 1 kb) cis-regulatory elements that facilitate transcription of nearby genes. Enhancers were initially defined not by their genomic features, but by their ability to enhance transcription irrespective of distance, position and orientation relative to a target gene [1–4]. Enhancers are a common feature of metazoan genomes far outnumbering protein-coding genes, with estimates of over a million enhancers in the human genome [5]. The sheer number of enhancers present in the genome suggests an incredible complexity of combinatorial gene regulation. Indeed, studies over the years have demonstrated that genes receive regulatory input from multiple enhancers whose usage is regulated in space and time over the course of development [6]. Further, these elements drive spatio-temporal changes in gene expression resulting in morphological divergence among closely related species [7–9].

Enhancers are thought to function by bridging components of the transcription machinery to target protomers, facilitating transcriptional ‘bursting’ and transcription elongation [10,11]. While enhancers tend to regulate nearby genes, an enhancer can be located up to a megabase from its target gene, potentially regulating any number of genes in between. A prevailing theory over the past decade is that spatial, rather than linear, distance between enhancers and promoters controls enhancer–promoter cooperativity, and this notion is supported by a number of genomics and microscopy-based techniques [12]. However, location in space is not sufficient to explain enhancer activity and specificity [13]. Additionally, functional reporter assays for enhancer activity reveal a complicated logic between enhancers and promoters, suggesting that sequence determinants also play a role in enhancer–promoter cooperativity [14,15]. Further, enhancers themselves are known to function cooperatively *in vivo* and may have additive effects on their target genes [16], thus complicating interpretation of functional studies attempting to couple a single enhancer to its target promoter. Thus, greater experimental insight is needed to refine predictive models of enhancer–promoter interaction and function.

Excellent reviews have covered determinants of regulatory element activity [17], how enhancer–promoter interactions are established and maintained [12], and transcription factor function at enhancers [18]. Additionally, enhancers can be defined by their chromatin features, including high DNA accessibility and a unique chromatin signature including both histone methylation and acetylation [19]. Here, we focus on the molecular mechanisms that regulate the chromatin states at enhancers and discuss recent studies regarding the impact of these chromatin states on enhancer function. We also discuss how enhancer chromatin may play a role in novel theories of transcription such as the role of biomolecular condensates in gene regulation.

## 2. Features of enhancer chromatin

**Figure 1.**
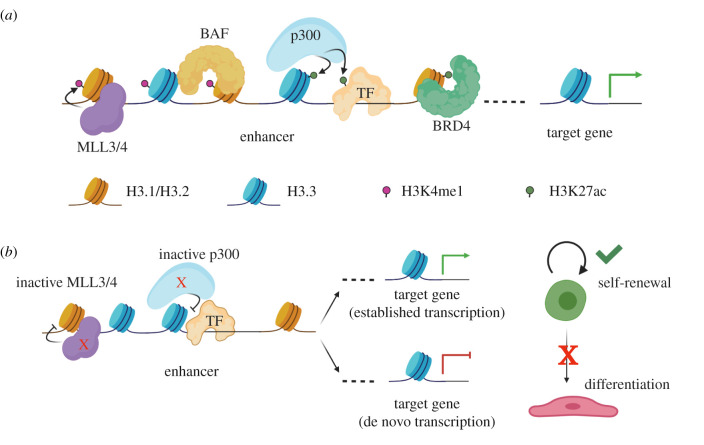
Features of enhancer chromatin. (*a*) An active enhancer bound by transcription factors (TF) and enriched in H2A.Z/H3.3 nucleosomes (blue), H3K4me1 (magenta) and H3K27ac (green). Histone methyltransferases MLL3/4 catalyse mono-methylation of H3K4, while acetyltransferases CBP/p300 acetylate both histones and transcription factors. (*b*) Catalytic activities of MLL3/4 and p300 are dispensable for maintaining transcription in embryonic stem cells (green) but are required to drive transcription upon stress response or differentiation. Created with BioRender.com.

Active cis-regulatory elements exhibit distinct chromatin features, including relatively low nucleosome occupancy, reflected by DNase hypersensitivity, and corresponding engagement by transcription factors and transcriptional coactivators (figure 1*a*). These regions are enriched with the histone variants H3.3 and H2A.Z [20,21], both of which have been associated with reduced nucleosome stability and dynamic nucleosome exchange [22,23]. In addition, nucleosomes at enhancers carry specific histone post-translational modifications, including histone H3 mono-methylated at Lys4 (H3K4me1) and histone H3 acetylated at Lys27 (H3K27ac) [24]. This signature was originally identified by the ENCODE project as a global feature of active enhancers and is often used to systemically annotate these regions *a priori* in a wide variety of biological contexts [5,25,26]. H3K27ac is also present at active promoters, which can be distinguished from enhancers at the chromatin level based on their high levels of H3K4me3 compared with H3K4me1. In addition to H3K27ac, a modification catalysed by the histone acetyltransferases p300 and cAMP response element-binding protein (CREB) binding protein (CBP), regulatory elements carry a high level of lysine acetylation on both H3 and H4, including H3K9, H3K18, H3K64, H3K122, H4K5, H4K8 and H4K16 [27,28]. Enhancers are also marked by bidirectional transcription, and the nature and function of these short and short-lived enhancer RNAs (eRNAs) have been the subject of intense study in recent years [29].

## 3. Histone variants and chromatin accessibility

An accessible chromatin state is a defining feature of enhancers, presumably required for transcription factor and coactivator access to DNA that would normally be blocked by nucleosome formation (with the exception of so-called pioneer transcription factors, recently reviewed [30]). Nucleosomes at enhancers are enriched with the H2A.Z and H3.3 histone variants which are deposited at these regions by dedicated histone chaperones and chromatin remodelling complexes [20,21,23,31,32], although it must be noted that both H2A.Z and H3.3 also play roles at heterochromatic regions under certain circumstances [20,33–37]. Both H2A.Z and H3.3 are reported to contribute to reduced nucleosome stability, depending on the composition of the nucleosome [22,38]. Because of this, it has been hypothesized that the histone variant composition of the nucleosome may allow greater access to underlying DNA, thus positively contributing to transcription factor binding. While this seems to be the case for H2A.Z deposition, the relationship between H3.3 deposition and chromatin accessibility is more complicated, as discussed below.

### 3.1. H2A.Z

H2A.Z contains sequence differences from its replication-coupled counterpart H2A that may explain differences in nucleosome stability. Specifically, these two proteins differ in their C-terminal L1 loops which form H2A–H2A contacts within the nucleosome, resulting in steric occlusion in heterotypic H2A.Z-H2A containing nucleosomes that reduces nucleosome stability [39]. H2A.Z also differs from H2A in its C-terminal docking domain, effectively reducing nucleosomal contacts with the H3-H4 dimer [40]. Further, H2A.Z undergoes step-wise deposition by the SWI/SNF chromatin remodelling proteins, SRCAP and p400, followed by nucleosome eviction by the H2A.Z-specific chaperones, INO80 and ANP32E, at actively transcribed regions [41–44]. Collectively, both the distinct biophysical properties and the dynamic remodelling of H2A.Z containing nucleosomes at regulatory elements is hypothesized to provide an excellent ‘window of opportunity’ for transcription factor binding. In support, biophysical experiments demonstrate that H2A.Z nucleosomes experience greater diffusion resulting in increased transcription factor binding to underlying DNA [45,46] and that H2A.Z nucleosomes lower barriers for DNA engagement by transcription machinery [47]. These *in vitro* observations agree with observations made in the more complex environment of the nucleus, where studies demonstrate that H2A.Z is required both for maintaining open chromatin states and binding of the pluripotency transcription factor, Oct4, during ESC self-renewal [36], and also for chromatin remodelling resulting in nucleosome eviction and subsequent gene activation during ESC differentiation [48].

### 3.2. H3.3

In contrast to the considerable sequence differences between H2A.Z and H2A, H3.3 differs from replication-coupled H3 by only a few amino acids and H3.3 containing nucleosomes are essentially structurally identical to H3 containing nucleosomes [49]. Regardless, many studies show that regions enriched with H3.3 undergo more dynamic nucleosome exchange than other regions of the genome [50–56]. While these results might suggest that H3.3 is required to maintain chromatin accessibility at enhancers, nucleosome occupancy at regulatory elements does not change and chromatin does not become less accessible at enhancers in ESCs lacking H3.3 [53,57]. It may be that the nature of complexes formed on chromatin at sites of H3.3 deposition plays a greater role in mediating chromatin accessibility than H3.3 itself. For example, H3.3-enriched regions are highly enriched with transcription factor binding and other transcriptional machinery, including chromatin remodelling complexes [58–62]. H3.3 has long been known as a replacement histone variant [63,64], and it is possible that H3.3 functions not to promote nucleosome dynamics but is deposited at distinct regions as a result of nucleosome eviction. In support, HIRA, the chaperone responsible for H3.3 deposition at regulatory elements, is thought to be recruited to ‘naked’ RPA-coated DNA and functions by a ‘gap-filling’ mechanism to promote nucleosome occupancy [59,65]. Further, this hypothesis is in agreement with the fact that, unlike H2A.Z, no dedicated machinery responsible for H3.3 eviction has been identified to date. Interestingly, one recent study demonstrates that H3.3 can be recycled at regulatory elements by a specific composition of the HIRA chaperone complex involving the general H3-H4 chaperone, Asf1 [66,67], and thus may contribute to the maintenance of chromatin post-translational landscapes in this manner.

While H3.3 deposition does not appear to be required for maintaining chromatin accessibility at active enhancers, it does play a role in maintaining the post-translational modification landscape at these regions. Specifically, H3.3 deposition is required to maintain high levels of H3K27ac at active enhancers and promoters in mESCs [57,62] and global levels of H4K16ac in NSCs [68]. Interestingly, neither H3K4me1 at enhancers nor H3K4me3 at promoters is affected by loss of H3.3, in line with findings that H3K4me1 is upstream of H3K27ac at enhancers (see below) [69,70]. The striking interpretation of this result together with data discussed above is that, once established, high levels of H3K27ac are not required to maintain chromatin accessibility at active enhancers [57]. This interpretation is supported by a recent study demonstrating little change in chromatin accessibility at enhancers in cells treated with a chemical CBP/p300 bromodomain inhibitor that results in global decreases in H3K27ac [71] and by a notable study in which mutation of endogenous H3.3K27 to H3.3K27R greatly reduced H3K27ac levels without a corresponding decrease in chromatin accessibility at enhancers [72]. Of note, we find that regions that maintain accessible chromatin when H3K27ac levels are reduced are highly enriched with lineage-specific transcription factors [73], suggesting that once established, gene regulatory networks may be self-sustaining without the need for high levels of histone acetylation [74].

## 4. Histone modifications and enhancer activation

### 4.1. H3K4me1

H3K4 methylation was first identified as a mark of active chromatin due to its enrichment in the transcriptionally active *Tetrahymena* macronuclei [75]. Studies in yeast and mammalian cells revealed that H3K4 can be mono- di- or tri-methylated, and that these marks were not interchangeable [76–78]. In a landmark study, H3K4me1 was genomically linked to active enhancers while H3K4me3 was found to be present at active promoters [79]. While these signatures are now routinely used to define regulatory elements, it is important to note that (i) H3K4me1 covers broad regions and is enriched not only at enhancers, but also at promoters and into the gene body [80,81], and (ii) H3K4me3 can be detected at highly active enhancers [82], in line with the model that multiple rounds of transcription contribute to high levels of H3K4me3 at promoters [83].

H3K4 is methylated in mammalian cells by the MLL/Set1 family containing six histone lysine methyltransferases [84,85]. Four of these proteins, Set1a and Set1b (homologous to *Drosophila* Set1) and MLL1 and MLL2 (homologous to *Drosophila* Trithorax [Trx]), are generally considered to facilitate H3K4me3 at promoters under various contexts [86,87]. Studies in recent years demonstrate that the remaining family members, MLL3 and MLL4 (homologous to *Drosophila* Trithorax-related [Trr]) are responsible for the majority of H3K4me1 at enhancers [88–90]. These findings have been confirmed in a number of model systems, including adipogenesis, cardiac development, lymphogenesis and activation of macrophages [90–93].

Generally, histone-modifying enzymes do not contain sequence-specific DNA binding domains, so their association with transcription factors should serve as a recruitment mechanism for their placement on chromatin. As such, it is not surprising that several studies report that transcription factor binding promotes MLL recruitment and subsequent H3K4me1 at enhancers [94–96]. Based on both developmental and genetic studies, it is generally accepted that H3K4me1 is upstream of H3K27ac at enhancers. Many developmental enhancers show evidence of ‘priming’ or ‘poising’, in which the region is enriched with H3K4me1 but not H3K27ac in the absence of detectable transcription [97–102]. Further, H3K4me1 often appears prior to H3K27ac during differentiation [90,103,104] and inducible transcription [103,105]. H3K27ac levels are also reduced upon loss of MLL3/4 activity, suggesting that H3K4me1 is upstream of H3K27ac at regulatory elements [69,70,89,90]. It is unclear how H3K4me1 primes enhancers for subsequent p300 activity. While reduced CBP/p300 recruitment has been reported upon MLL3/4 deletion [106], poised developmental enhancers are both enriched with H3K4me1 and bound by p300 without evidence of its enzymatic activity [97,98]. Thus, additional studies are needed to address the interplay between MLL3/4, p300, and their respective activities at enhancers.

It is well-documented that histone post-translational modifications can act as scaffolds to recruit chromatin-associated proteins to specific genomic regions (figure 1*a*). While initial studies identified mainly proteins whose recruitment to chromatin was mediated via interaction of PHD domains with H3K4me2 or H3K4me3 [107], there is evidence that certain PHD domains can also recognize lower-order methylation or even unmethylated lysines [108]. In fact, ZMYND8 has been identified as a PHD domain-containing chromatin factor at enhancers whose recruitment may be stabilized by the presence of unmethylated or monomethylated H3K4 [109–111]. Recent evidence suggests that H3K4me1 may also contribute to complex formation on chromatin through interaction with the PHD1–PHD2 domains of BAF45C, a member of the BAF chromatin remodelling complex [112]. ESCs deleted of MLL3/4 showed reduced BAF complex recruitment to enhancers, with structural studies demonstrating that the PHD1 domain of BAF45C recognizes H3K14ac and the PHD2 domain recognizes H3K4. The binding pocket in PHD2 can accommodate unmodified H3K4 or H3K4me1 while sterically disfavouring H3K4me2 or H3K4me3, however mechanisms that promote selectively of H3K4me1 over unmodified H3K4 remain unclear. *In vitro*, H3K4me1 nucleosomes were better substrates for the BAF complex in a chromatin remodelling assay compared with unmodified nucleosomes or nucleosomes containing higher-order H3K4 methylation (e.g. H3K4me2, H3K4me3) [112]. Further, H3K4me1 was shown to be important for maintaining enhancer promoter contacts in ESCs and for establishing new enhancer–promoter contacts during differentiation [113], suggesting that the local chromatin modification state plays an important role in nucleosome positioning and 3-dimensional interactions between regulatory elements.

Despite its abundance, and the mechanistic insights into H3K4me1 described above [112,113], several recent studies suggest that H3K4me1 is not necessary for maintaining enhancer activity under steady state conditions (figure 1*b*). Interestingly, mutations that disrupt MLL3/4 catalytic activity are generally less detrimental than loss of MLL3/4 protein, suggesting that MLL3/4 may play a more important structural role at enhancers rather than any requirement for its enzymatic activity. For example, deletion or mutation of the catalytic SET domains of MLL3 and MLL4 has minor effects on transcription despite abolition of H3K4me1 from enhancers [69,70], and Trr catalytic activity is dispensable for *Drosophila* development [69]. However, deletion of MLL3/4 in embryonic stem cells (ESCs) results in reduced Pol II occupancy and transcription at both enhancers and genes, and loss of *trr* in *Drosophila* is embryonic lethal [69,70]. The implication of these results is that MLL3/4 facilitates transcription independently of its catalytic activity and through an as yet unknown structural function.

Why, then, has MLL3/4 catalytic activity and the H3K4me1 mark persisted through evolution? Are there contexts in which it is necessary? Or does MLL3/4 play only a structural role at enhancers? The chromatin state of enhancers is dynamic, to ensure both proper development and transcriptional response to environmental stimuli. It is therefore possible that H3K4me1 is not required for proper differentiation, but may be necessary for stress response [114]. It is worth bearing in mind that MLL3/4 KO mESCs exhibit differentiation defects [90,103] and that loss of MLL3/4 has a greater impact on transitions between ground state and naive pluripotency than loss of its catalytic activity [115]. While catalytic mutation of MLL4 causes embryonic lethality in mice, mutation of the MLL4 active site destabilizes the protein, making it difficult to identify the cause of lethality [116]. *Drosophila* embryos expressing inactive Trr develop normally at room temperature, but exhibit subtle developmental defects at elevated temperatures [69]. Thus, while MLL3/4 protein appears to be essential for maintaining transcription, H3K4me1 may be required for a transcriptional response to acute stress, potentially by recruiting the chromatin remodelling BAF complex and/or stabilizing promoter–enhancer contacts [112,113].

It is also important to note that the key study regarding developmental function of MLL3/4 catalytic activity was carried out in *Drosophila*, a model organism that does not use DNA methylation as a genomic regulatory mechanism. Interestingly, H3K4 methylation prevents chromatin recognition by DNMT3 L, a regulatory subunit associated with both de novo methyltransferases DNMT3a and DNMT3b [117]. While there have been reports of a role for LSD1 in H3K4me1 demethylation during developmental enhancer decommissioning [115,118], several studies suggest that H3K4me1 is maintained at enhancers that are used late in development, even after the enhancer shows no signs of transcriptional activity [106,119]. Interestingly, enhancers typically show low levels of DNA methylation [120–122], and such ‘vestigial’ enhancers are protected from DNA methylation even into adulthood and thus serve as a ‘fossil record’ of gene activity [119,121,123]. The cellular logic behind this remains unclear, as these regions are more susceptible to gene dysregulation after prolonged PRC2 deficiency [119]. Of note, this study focused on the intestinal epithelium, one of the most rapidly self-renewing tissues in the body with the ability to recover lost stem cells through dedifferentiation of downstream lineages [124,125]. Perhaps maintaining a cellular record of developmental tissue-specific enhancers promotes such regeneration.

### 4.2. H3K27ac

Lysine acetylation was first observed on histones more than 50 years ago [126]. Over the following decades, it was revealed that hyperacetylated histones are abundant near active genes, while heterochromatic regions are hypoacetylated. Early studies of histone acetylation suggested that it facilitates DNA accessibility by neutralizing positively charged lysines, thereby reducing their affinity to DNA [127–131]. A direct link between histone acetylation and transcription was established decades later in the 1990s, with the purification of histone acetyltransferase A (HAT A), a homologue of the yeast transcriptional coactivator Gcn5p, from *Tetrahymena* macronuclei [132]. This breakthrough led to an explosion of interest in histone modifications, leading to the identification of more than a dozen HATs that acetylate all four histones in a variety of contexts [133]. Further, it is now clear that in addition to the charge neutralization model described above, lysine acetylation on histones acts as a scaffold to recruit proteins containing bromodomains, YEATS domains, and tandem PHD domains to chromatin, and that many transcription-related proteins and complexes contain such domains [134].

Active enhancers and promoters recruit p300 and CBP, two closely related coactivators collectively termed CBP/p300 due to their high degree of sequence similarity (figure 1*a*). p300 and CBP are both large approximately 300 kDa proteins with multiple DNA-binding and histone-interacting domains. Interestingly, the CBP/p300 histone acetyltransferase (HAT) domain lacks homology with that of other HAT families such as the GNAT and MYST HAT families [135]. To date, p300 and CBP are the only HATs known to catalyse the H3K27ac modification characteristic of active enhancers [135]. A recent study demonstrates that p300 specificity for H3K27 is dictated by interaction of its ZZ-type zinc finger domain with the first six N-terminal amino acids of the H3 tail [136], which may serve as a ‘molecular ruler’ to position the HAT domain at H3K27. In addition to H3K27, CBP/p300 acetylate additional lysines on histone proteins (e.g. H3K14, H3K18 and H3K23) as well as a number of non-histone nuclear proteins, many involved in transcriptional regulation [137]. p300 and CBP are both expressed in all tissues and required for development [138,139], but they are not interchangeable. They have distinct specificities toward each substrate lysine *in vitro* [140,141], and each HAT performs distinct tissue-specific roles [73,142–147]. Strikingly, while CBP and p300 exhibit near identity of amino acid sequence in conserved domains, a much higher degree of variability exists outside of these regions, in parts of the proteins that are predicted to be unstructured. As these regions are likely to engage in protein–protein interactions, it is intriguing to speculate that they may engage different protein binding partners that dictate differential CBP and p300 acetyltransferase activity in a cell-type-dependent manner.

Since the advent of genome-wide ChIP assays, H3K27ac has emerged as the conventional signpost to identify putative enhancers. Despite the predictive power of this modification, its precise role in gene regulation has been difficult to pinpoint. Enhancers and promoters are enriched in acetylation at several lysine residues, and it remains unclear if and when a particular acetylation mark, or combinations thereof, serve a specific function. This challenge arises in part from the intractability of histones to genetic manipulation. Mammalian histones are encoded in multiple large repeating arrays [148], all of which would need to be precisely mutated to perturb a particular acetylation or set of acetylations. However, such elegant studies have been performed in *Drosophila*, complementing deletion of its single histone cluster with plasmid-based tandem transgene copies to introduce histone mutations in the context of a multicellular organism [149,150]. While, thus far, these studies have mainly focused on the role of histone modification in gene silencing, interesting inferences about gene activation have been made in cases in which an amino acid can be associated with either depending on the nature of the modification (e.g. H3K27ac is associated with gene activation whereas H3K27me3 is associated with gene silencing). For example, several studies have used H3K27R mutants to study the function of polycomb activity, and indirectly, these studies infer that gene activation can occur in the absence of H3K27ac [149,151,152]. However, these mutations were made in animals expressing wild-type H3.3 (or in one case, an H3.3 mutation was made in animals expressing wild-type H3), leaving open the possibility that H3K27ac function is not specific to the variant of H3 that is acetylated [57].

**Figure 2.**
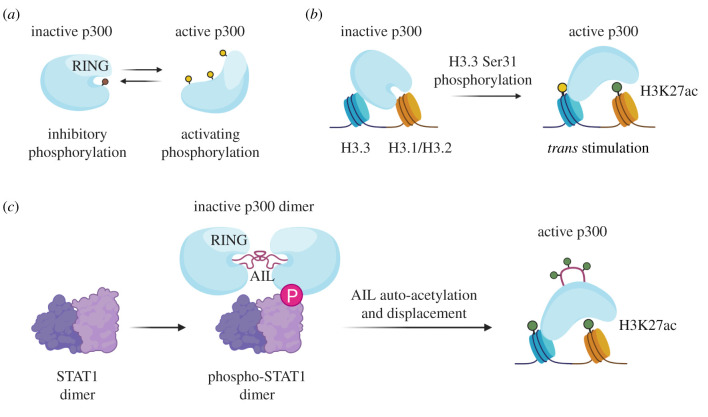
Regulation of histone acetyltransferase CBP/p300 activity. (*a*) p300 is subject to activating (yellow—S1834, S2271, S2279, S2291, S2315) and inhibitory (brown—S89) phosphorylation. Upon activation, p300 undergoes a conformational change to displace the RING domain and expose the active site. (*b*) Phosphorylation (yellow) of Ser31 of H3.3 (blue) nucleosomes stimulates p300 activity (H3K27ac—green) on canonical histones (orange). (*c*) Stimulus-induced phosphorylation of STAT1 induces p300 dimerization and auto-acetylation (green) of its auto-inhibitory loop (magenta), resulting in p300 activation.

Several recent studies have called into question the conventional wisdom about the necessity of H3K27ac for maintaining steady state transcription. Loss of MLL3/4 catalytic activity in ESCs results in a global reduction in H3K27ac levels, but has modest effects on transcription (figure 1*b*) [69,70]. Similarly, we and others have found that loss or mutation of H3.3 (both H3.3K4A and H3.3K27R) reduces H3K27ac with little effect on mESC self-renewal and transcription (figure 2*b*) [57,62,72]. It is important to note that H3K27ac is not completely abolished in any of these experimental models and it is possible that H3K27ac has not been reduced below its functional threshold, particularly given the nutrient-rich environment of *in vitro* culture conditions. Also, it is also important to recognize that CBP/p300 acetylate many lysines, both on histone and non-histone proteins [137], and it is likely that it is this broad network of acetylation that contributes to gene expression. However, it is also possible that the chromatin-based mechanisms that promote gene activation are different than those that are required to maintain ongoing transcription under steady state. As noted above, regions that seem impervious to loss of H3K27ac with respect to chromatin accessibility and transcriptional output tend to be highly bound by cell-type-specific transcription factors [73], and it is possible that this established state obviates the need for high activity from coactivators and high levels of acetylation [74,153]. Interestingly, while all of the above studies used mESCs as a model, only two used differentiation as a model to induce new transcription programmes. In both cases, H3.3K4A mutant ESCs (that results in reduced H3.3 stability) and H3.3 KO mESCs showed delays or defects in the ability to transcribe new genes [57,62], probably due in part to an inability to install H3K27ac at latent enhancers [57]. These data support a model in which high levels of H3K27ac are dispensable for maintaining transcription but important for responding to developmental stimuli.

## 5. Mechanisms of CBP/p300 activation

Given its role in developmental gene regulation, it is not surprising CBP/p300 responds to local signalling environments and that its enzymatic activity can be modulated by kinase activity and phosphorylation (figure 2*a*). For example, protein kinase C (PKC)-mediated p300 Ser89 phosphorylation inhibits p300 catalytic activity [154], while phosphorylation by, Akt (S1384) and mTORC1 (4 Ser residues) stimulates p300 activity in a variety of biological contexts [155–157]. While it is unclear what specific structural changes most of these modifications induce, there is evidence that they promote allosteric activation of p300 through dissociation of the HAT domain and the auto-inhibitory RING domain, which blocks substrate access to the active site [157]. Phosphorylation of CBP/p300 regulates not only its catalytic activity, but also its interactions with TFs and recruitment to chromatin. For instance, the phosphorylation state of CBP determines its mutually exclusive binding to p53 or NF-κB [158]. Additionally, phosphorylation of CBP at Ser 436, a residue with no equivalent in p300, regulates its recruitment to Pit-1 and AP-1 response elements [159]. This unique phosphorylation suggests distinct modes of regulation for CBP and p300, adding support to the hypothesis that CBP and p300 are not always interchangeable.

Several recent studies demonstrate that, in addition to its own phosphorylation, p300 is enzymatically activated in response to the phosphorylation of other proteins present at enhancers, thus both directly and indirectly responding to signalling events that culminate on chromatin. For example, one study found that phosphorylation-mediated dimerization of transcription factors led to a rapid increase in p300 activity towards histone substrates [160]. Previous studies have shown that several regions of p300 are autoinhibitory, for example the RING domain prevents effective substrate access to the HAT domain [161]. In addition, the lysine-rich, intrinsically disordered autoinhibitory loop (AIL) acts as a ‘pseudosubstrate’ that inhibits the acetylase activity of adjacent p300 molecules in *trans* [162]. Interestingly, transcription factor dimerization promotes p300 *trans*-autoacetylation of lysines in the AIL, thus neutralizing this positively charged loop and resulting in allosteric activation of the enzyme (figure 2*c*) [160]. A similar mechanism of charge-based AIL displacement was recently proposed to explain activation of CBP by negatively charged eRNA [163]. Together with the studies described above, these data suggest that p300 PTMs, both acetylation and phosphorylation, may serve the common purpose of facilitating allosteric changes that regulate access to the p300 active site.

Several recent studies also demonstrate a link between H3.3 phosphorylation and p300 activity at enhancers [57,164]. Interestingly, the N-terminal tails of canonical H3 and H3.3 differ by only one amino acid. While H3.1 and H3.2 contain an alanine at position 31, H3.3 has a unique and highly conserved serine that has been reported to be phosphorylated by both checkpoint kinase 1 (Chk1) and Aurora B [165–167]. A study from the Almouzni lab demonstrates that a phosphomimetic mutant of H3.3 at this position (H3.3S31D) results in increased H3K27ac in *cis* in both human cell lines and Xenopus embryos [164]. Our own work demonstrates that phosphorylation of H3.3 Ser31 (H3.3S31ph) facilitates p300 activity not only on phosphorylated tails, but also in *trans* on canonical H3 substrates in mESCs (figure 2*b*) [57]. In our model, H3.3 deposition and signalling-mediated phosphorylation serves as a catalyst to globally promote the enzymatic activity of p300 that is already bound at enhancers. This observation could be explained by several mechanisms, for example: (i) H3.3S31ph may recruit a p300 activator or prevent the binding of an inhibitor at enhancers; (ii) H3.3S31ph allosterically stimulates p300 activity, possibly by forming a stable interaction in a manner analogous to stimulation by eRNA (although the negative charge provided by H3.3S31ph is much less than that of an eRNA, and to date, there is no structural evidence of stable interaction between p300 and the H3 tail [168]); or (iii) phosphorylated H3.3 may be a more permissive substrate which, upon acetylation, allows p300 to remain chromatin-bound via its bromodomain to acetylate adjacent canonical histones. Interestingly, H3.3S31ph has previously been linked to gene activation [169] and another recent study reported structural evidence that H3.3S31ph promotes the histone methyltransferase activity of SETD2 towards H3K36 during macrophage stimulation [170]. Taken together, these findings suggest that one function of the highly conserved serine at position 31 on H3.3 is to influence chromatin states at active regulatory elements and genes.

## 6. Recognition of histone acetylation

As mentioned above, one likely function of enhancer acetylation, both on histones and transcription factors, is to serve as a scaffold for recruitment of transcription machinery to chromatin. Acetylated lysines mainly on histone, but also non-histone proteins, are recognized by bromodomains, which are present in a diverse array of transcriptional coactivators [134]. These include HATs themselves (e.g. CBP/p300, PCAF, GCN5 among others) which may have evolved to reinforce their own products, ATP-dependent chromatin remodellers (e.g. SWI/SNF complex members SMARCA4—also known as BRG1—and SMARCA2—also known as BRM, PBAF complex members polybromo 1 and BRD7, ISWI complex member BPTF, the BAZ family of proteins), general transcription factors (e.g. TAF1), and the bromodomain and extraterminal domain (BET) family of proteins involved in productive RNAPII elongation (e.g. BRD2, BRD4) (figure 1*a*). Bromodomain-containing proteins are anchored to chromatin through their association with acetylated lysine, and then in turn act as scaffolding proteins to recruit additional transcriptional machinery. For example, BET proteins through their extra terminal (ET) domain recruit the positive elongation factor b (P-TEFb) to promoters, resulting in cyclin-dependent kinase 9 (CDK9) phosphorylation of RNAPII and pause release [171–173]. The presence of bromodomains in proteins involved in all stages of recruitment suggests that acetyllysine recognition must play a critical role in these processes, and that nucleation of these complexes at enhancers and promoters [174–176] results in a feed-forward loop that stabilizes transcription. In fact, the act of transcription may in turn further stabilize these complexes, as recent reports demonstrate that eRNAs can both stimulate CBP activity and H3K27ac and stabilize subsequent binding by BRD4 [163,177].

In addition to bromodomains, both tandem plant homeodomain (PHD) zinc fingers and Yaf9, ENL, AF9, Taf14, Sas5 (YEATS) domains have recently emerged as additional classes of lysine acetylation readers [178–180]. While the tandem PHD domains also recognize unmodified lysines and are thus unlikely to be involved in complex recruitment to chromatin, the YEATS domain-containing proteins are bonafide acyl-lysine readers and are associated with chromatin-remodelling complexes or transcription-associated complexes and mainly linked to transcription elongation [178]. Interestingly, the YEATS domain binding pocket is slightly larger than that of the bromodomain and is able to accommodate longer chain acylations compared with acetylation [180–183]. In fact, since its discovery as an H3K9ac reader, the YEATS domain has been found to bind to crotonylated lysines with higher affinity than acetylated lysines [181,182]. Like acetylation, higher-order acylations such as crotonylation are enriched at regulatory elements such as enhancers and promoters [184]. While crotonylation has been linked to changes in transcription related to cellular metabolism [185,186], the direct role of higher-order acylations in enhancer regulation remains to be discovered.

## 7. Role of enhancer chromatin in phase separation of transcription machinery

**Figure 3.**
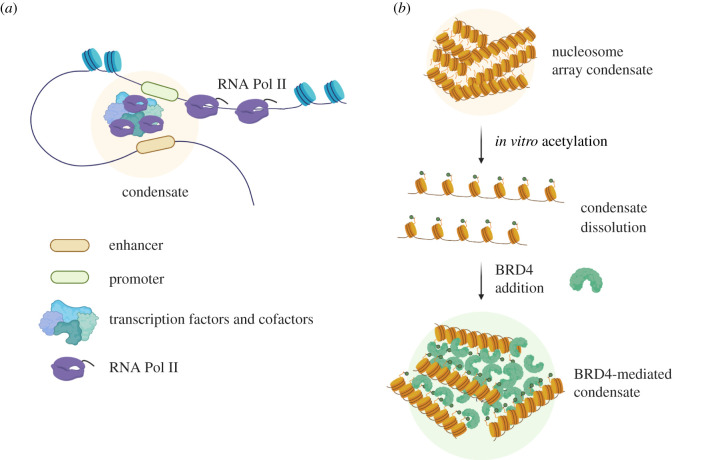
Phase separation of transcription machinery. (*a*) Transcription factors and cofactors with intrinsically disordered regions drive the formation of biomolecular condensates at enhancers. (*b*) Hypoacetylated nucleosome arrays assembled *in vitro* spontaneously undergo liquid–liquid phase separation (LLPS). Acetylation by p300 blocks LLPS, while addition of synthetic multi-bromodomain proteins (green) restores biomolecular condensates.

Although there are thousands of enhancers in any given cell type with signatures of active chromatin, a subset of enhancers display particularly high density of transcription factor binding, are highly enriched with cofactors such as CBP/p300, resulting in high levels of H3K27ac, and are bound by high levels of chromatin regulators such as MED1 [187,188]. These regions, often composed of many individual enhancers and called super-enhancers (SEs), are in close contact with the promoter of the gene they regulate [189,190] and are thought to play important roles in cell-type-specific processes and have been implicated in both development and disease [187,188,191,192]. There is mounting evidence that such enhancers and promoters may undergo liquid–liquid phase separation (LLPS), forming ‘biomolecular condensates’ with high concentrations of transcription machinery [193] (figure 3*a*). Such structures are hypothesized to form hubs where multiple enhancers and promoters converge resulting in a high frequency of transcription with low variation [194].

Biomolecular condensates are thought to form through weak, multivalent, and dynamic interactions that can be facilitated by intrinsically disordered regions (IDRs) in proteins. Such IDRs are common in the activation domains of transcription factors as well as coactivators such as MED1 and BRD4, and these regions have been shown to promote LLPS *in vitro* and condensate formation in cells [195–198]. In this model, genomic specificity of condensate formation must be driven by some region with the ability to recognize specific DNA sequences, and indeed, most TFs contain such a DNA binding domain. In fact, high densities of TF binding sites, such as those found at SEs, are thought to promote condensate formation, suggesting that a combination of structured and dynamic interactions may drive phase separation in cells [197] (figure 3*a*).

In addition to IDR-based non-traditional protein–protein interactions, structurally based weak multivalent interactions may contribute to the formation of nuclear condensates. Interestingly, linked domains resulting in multivalent engagement are a common feature of chromatin-associated proteins and complexes [199]. For example, BAF180 (also known as polybromo), a member of the PBAF chromatin remodelling complex, contains six bromodomains, and the core complex members of the NURF chromatin remodelling complex contain at least 10 domains that engage with chromatin. Based on these observations, one logical hypothesis is that the chromatin landscape at enhancers contributes to condensate formation at these regions by promoting a local environment enriched with high concentrations of weakly associated proteins. Interestingly, a recent study found that recombinant chromatin itself exhibits condensate properties *in vitro* in the absence of cofactors enriched in IDRs, and that this property requires the unstructured histone tails that protrude from the nucleosome core [200]. Strikingly, the authors found that acetylation by p300 prevents phase separation, while addition of multi-bromodomain proteins to acetylated chromatin generates a distinct condensate which fails to fuse with condensates containing unmodified chromatin, again supporting the idea that multivalent interactions are important for this process (figure 3*b*).

Ultimately, what is the purpose of condensate formation at cell-type-specific enhancers? A number of studies demonstrate that RNAPII assembles at condensates in cells [201–203], and the prevailing model is that this local accumulation of likely hundreds of copies of RNAPII allows seamless production of transcripts from important genes [10,11,202,204,205]. Interestingly, RNAPII must exit the condensate for productive elongation, and this process is regulated by phosphorylation of its highly repetitive and disordered C-terminal domain (CTD), effectively resulting in partitioning of RNAPII from one condensate to another [206–209].

## 8. Concluding remarks

In the past decade, major advances in DNA sequencing technology and computational tools have made it possible to identify a wide variety of epigenomic features genome-wide. However, identifying the function of each feature remains a challenge due to widespread cross-talk between histone variants, modifications and nucleosome depletion. For example, loss of either MLL3/4 activity or H3.3 deposition reduces H3K27ac levels without any clear effect of H3.3 on MLL3/4 activity. Together with the observation that targeted recruitment of dCas9-p300 to silent enhancers and promoters is sufficient for gene activation [210], these data suggest that H3K27ac may be downstream of other enhancer features such as open chromatin, transcription factor binding and methylation of H3K4. Interestingly, mounting evidence suggests that H3K4me1 and H3K27ac, the canonical PTMs associated with active enhancers, are dispensable for proper genome function in certain cellular or organismal contexts [57,69,70]. These observations raise the possibility that certain histone modifications may be incidental products of enzymes which perform necessary modifications on other chromatin factors. An important recent study found that CBP/p300 acetylates more than 200 transcription factors and cofactors [137] and, so far, few of these modifications have been investigated. Understanding the biological significance, if any, of these modifications will provide insights into unexplored roles of CBP/p300.

Emerging technologies will no doubt remain key to understanding enhancer biology. Novel imaging techniques will reveal the spatio-temporal dynamics of enhancer–promoter interactions in living cells, allowing greater understanding of how and when proximity is required developmentally [211,212]. Improved genomic approaches allowing resolution at the single-cell level coupled with elegant genetic and pharmacological perturbation techniques will increase our understanding of how and when chromatin states are established and decommissioned developmentally [213,214]. New protocols to perform multiple ‘omics’ experiments at the single cell level will result in greater understanding of the relationship between chromatin states and transcriptional output [215,216]. Concepts such as phase separation will provide a theoretical framework for integrating many new and old observations of the physical properties of proteins involved in transcription regulation [194]. Despite the great importance of such technological and theoretical advances, there is still much to be learned of the contribution of chromatin states to enhancer function using the tools of genetics, biochemistry and molecular biology. Using both novel and traditional methods, we expect many such important discoveries to be made in years to come.

## Data accessibility

This article has no additional data.
